# TLR3 expression correlates with apoptosis, proliferation and angiogenesis in hepatocellular carcinoma and predicts prognosis

**DOI:** 10.1186/s12885-015-1262-5

**Published:** 2015-04-09

**Authors:** Ming-Ming Yuan, Yu-Yin Xu, Li Chen, Xing-Yu Li, Jing Qin, Ying Shen

**Affiliations:** 1 Department of Pathological Anatomy, Nantong University, Qixiu Road 19, Nantong City, Jiangsu 226001 China; 2 Department of Pathology, Nantong Rich Hospital, Jiangsu, China; 3 Department of Nephrology, Affiliated Hospital of Nantong University, Nantong, Jiangsu China

**Keywords:** TLR3, HCC, Proliferation, Apoptosis, Angiogenesis, Prognostic

## Abstract

**Background:**

Toll-like receptor 3 (TLR3) plays a key role in innate immunity. In the present study, we analyzed tissues of patients with human hepatocellular carcinoma (HCC) to determine the significance of the relationship between TLR3 expression and cell proliferation, apoptosis, hepatitis B virus infections, angiogenesis and prognosis.

**Methods:**

We collected paraffin-embedded tissues from 85 patients with HCC who had complete histories and were followed for >5 years. The expression and intracellular localization of TLR3 and downstream proteins (TRIF, NF-κB, and IRF3) were detected using immunohistochemistry. Further, we determined the expression of proteins that mediate cell proliferation (Ki67, cyclin D1), apoptosis (survivin, bcl-2, caspases 3, 8, and 9), and angiogenesis (CD34, MMP-2) as well as the HBV proteins HBsAg and HBcAg. Apoptosis in HCC tissues was detected using TUNEL. We conducted dual-labeling immunohistochemical analyses of TLR3 expression and TUNEL activity.

**Results:**

TLR3 expression was significantly lower in HCC tissues compared with adjacent tissues. TRIF, NF-κB, and IRF3 correlated positively with TLR3 expression. Survivin and Bcl-2 expression correlated negatively with TLR3. The frequencies of caspases 3, 8, and 9 expression correlated positively with TLR3 signaling proteins. Cytoplasmic TLR3 and serum levels of HBsAg correlated positively. The apoptotic index determined using the TUNEL method and correlated positively with TLR3 expression. TLR3 expression in the cytoplasm correlated positively with TUNEL-positive cells and HBsAg. Ki67 and cyclin D1 correlated negatively with TLR3 expression. MMP-2 expression, microvessel density (CD34^+^) and endothelial progenitor cells (EPCs) correlated negatively with TLR3 expression. Kaplan–Meier survival analysis shows that TLR3 expression correlated with longer survival.

**Conclusions:**

The expression of TLR3 in HCC tissues may exert a synergistic effect on apoptosis and inhibit the proliferation of HCC cells, MMP-2 expression, generation of EPCs, and angiogenesis. Moreover, TLR3 expression may serve as a prognostic marker of HCC.

## Background

Hepatocellular carcinoma (HCC) is the sixth most prevalent cancer and the third most frequent cause of cancer-related deaths worldwide [1]. China has a high frequency of HCC, particularly in Qidong of Jiangsu Province, the site of this study. The prognosis of patients with HCC is generally poor, and frequent recurrence or metastasis after transplantation represents the main obstacle for long-term survival [2], which reflects the complexity and heterogeneity of HCC biology. Therefore, new approaches to address the mechanisms of HCC progression are required to develop effective prognostic techniques and to discover new therapeutic targets.

HCC is a hypervascular carcinoma, and angiogenesis, in which endothelial cells of pre-existing capillaries proliferate and migrate to form new vascular tips or so-called “vascular sprouts” or “endothelial buds” [3], plays an important role in the progression of HCC and contributes to its malignant phenotype, invasiveness, and high rates of recurrence and metastasis [4]. Solid tumors may not grow beyond 2–3 mm^3^ if vascular sprouts are blocked [3], suggesting that advances in research on vascular biology are paramount to the development of genetic engineering and proteomics technologies that may provide new and effective therapies for HCC that target angiogenesis.

The Toll-like receptor (TLR) family member TLR3 recognizes double-stranded RNA (dsRNA) of viruses, endogenous dsRNA released from dying cells, or synthetic dsRNA such as polyriboinosinic:polyribocytidylic acid (poly I:C). The TLR3 signaling pathway is mediated exclusively by the TRIF adapter, which is recruited to TLR3 by interaction between the TIR domains of the two molecules [5,6]. Various branches of the signaling pathway emanating from TLR3-TRIF lead to the activation of IRF3, NF-κB, and AP1 [7,8] and to the induction of apoptosis through pro-caspase-8 activation [9-11]. This pathway activates IRF3 and NF-κB, which act together to induce the production of antiviral IFNs and other cytokines [12]. Evidence indicates that the activation of NF-κB is mediated by RIP1 [13] and by TRAF6 in some cell types [14,15]. RIP1 and TRAF6 subsequently recruit TAB2 [16] and TAK1. TAK1 phosphorylates IKKα and IKKβ. IKKβ phosphorylates the NF-κB inhibitor IκB, eventually leading to degradation of IκB and the translocation of NF-κB to the nucleus [17].

TLR3 has attracted considerable attention from investigators in fields such as biochemistry, immunology, and medicine; however, we know little regarding the significance of TLR3 in human carcinoma cells. In the present study, we determined the expression and intracellular localization of TLR3 in patients’ HCC tissues and the relationship between the expression of TLR3 and cell proliferation, apoptosis, HBV infections, and angiogenesis. Our goal was to investigate whether activation of the TLR3 signaling pathway inhibits the growth of HCC.

## Methods

### Ethics statement

We certify that we have read Nantong University’s ETHICAL PRINCIPLES FOR CONDUCTING RESEARCH WITH HUMAN AND ANIMALS. The protocols for these studies were approved by the Ethics Committees of the Third People’s Hospital of Nantong and the Nantong Rich Hospital.

### Patient materials

The study group included 85 patients who were enrolled at the Third People’s Hospital of Nantong and the Nantong Rich Hospital between 2005 and 2009. We followed 76 patients after their discharge. The follow-up period ranged from 1 to 96 months (median 26 months). Tissues were excised from an area 2–3 cm distant from HCC nodules. Nontumor tissues were acquired from 15 liver-transplant donor specimens, 10 liver hemangioma specimens, and five liver trauma specimens. Patients underwent liver resection and were subsequently diagnosed histopathologically with HCC. The patients had clear records of serum AFP level, tumor size, and lymph node metastasis. The ethics committee of each institution approved this study, and all patients granted written informed consent.

### Tissue microarrays

Tissue microarrays (TMA) were prepared according to the method of Qun (Patent Number: ZL 2008 1 0022 170.4). Briefly, all HCC tissues were stained using hematoxylin and eosin and were reviewed by two histopathologists. Representative areas free from necrotic and hemorrhagic materials were marked in the paraffin blocks. Two cylindrical tissue cores (1.6-mm diameter) were removed from the donor blocks and transferred to the recipient paraffin blocks, and their array positions were recorded. Each TMA block contained over 100 cylinders, and the final TMAs comprised samples from 85 cases of HCC and 85 cases of adjacent nontumor tissues (ANT). Consecutive sections (4 μm thick) were cut from the array blocks and placed on adhesive microscope slides for immunohistochemical analysis.

### Immunohistochemical analysis

The Envision+/DAB analysis method was performed using formalin-fixed, paraffin-embedded 4-μm sections from all patients. Antibodies against 13 proteins were used to analyze the sections (Table 1). The sections were dewaxed in xylene and heated in a microwave oven. For antigen retrieval, slides were heated at 95°C for 10 min in sodium citrate buffer (10 mM sodium citrate monohydrate, pH 6.0). The slides were cooled for 20 min at room temperature and then incubated in Envision + peroxidase blocking solution (Dako Cytomation, Glostrup, Denmark) for 5 min and rinsed with 0.05% Tris-buffered saline (TBS)/Tween 20 buffer, pH 7.4. The slides were then incubated with primary antibodies for 30 min at room temperature. The slides were washed with 0.05% Tween 20 in TBS (pH 7.4). Detection was achieved with the DAKO Envision+/HRP system (DAKO, Carpinteria, CA, USA). The color was developed using a 15-min incubation with diaminobenzidine (DAB) solution (DAB kit IL1-9032) (Fuzhou Maixin Biotech. Co., Ltd., China), and sections were lightly counterstained with hematoxylin. Positive and negative controls (TBS was substituted for primary antibody at concentration of 1:200) were performed for each assay.

**Table 1:** **Antibodies used for immunohistochemical analyses**

Proteins	Manufacturer	Dilution	Positive
TLR3	ABCAM (AB12085)	1:50	Membrane, Cytoplasm
TRIF	ABCAM (AB101232)	1:50	Membrane, Cytoplasm
NF-κB	Cell signaling (SER536)	1:100	Cytoplasm, Nucleus
IRF3	ABCAM (AB76493)	1:50	Cytoplasm, Nucleus
Ki67	Fuzhou Maixin Biotech (RMA-5542)	1:50	Nucleus
CyclinD1	Fuzhou Maixin Biotech (RMA-0541)	1:50	Nucleus
Survivin	Fuzhou Maixin Biotech (RAB-0536)	1:50	Cytoplasm
Bcl-2	ABCAM (AB12085)	1:100	Cytoplasm
caspase 3	Cell signaling (ASP175)	1:50	Cytoplasm
Caspase 8	Cell signaling (ASP391)	1:50	Cytoplasm
Caspase 9	Proteintech (10380-1-AP)	1:50	Cytoplasm
CD34	Fuzhou Maixin Biotech (MAB-0034)	1:50	Membrane, Cytoplasm
MMP-2	Fuzhou Maixin Biotech (MAB-0244)	1:50	Cytoplasm
Two-step immunohistochemical staining kit	Fuzhou Maixin Biotech (KIT-9801)		

### Staining patterns and data evaluation

The staining patterns were defined as “P” (cytoplasm), “M” (membrane), and “N” (nucleus). The average optical density (OD) of the immunostained HCC cells of each sample was measured using a morphological analysis system (Image J). Staining was defined according to average OD as follows: strongly positive (++), 40–60; weakly positive (+), 20–39; and negative (−), 0–19.

CD34 was mainly expressed in a scattered pattern in endothelial cells of microvessels. The most densely stained zones were selected at the invading tumor front. We analyzed blood vessels with a clearly defined lumen or linear vessel shape, but not single endothelial cells. The mean vessel count obtained from five fields at × 200 magnification was used to define the mean vesicle density (MVD). The final EPC number is expressed as the mean of five fields.

### Terminal deoxynucleotidyl transferase-mediated dUTP nick end labeling (TUNEL)

A TUNEL kit (Promega, USA) was employed to detect apoptosis. Briefly, paraffin-embedded sections were deparaffinized and dehydrated. After washing in phosphate-buffered saline (PBS), sections were treated with 20 μg/mL of Proteinase K for 20 min. After washing in PBS three times (3 min each), sections were rinsed with 0.3% Triton X-100 for 10 min followed by washing in PBS. These sections were incubated with the TUNEL reaction mixture at 37°C for 1 h. After washing in PBS three times (3 min each), sections were treated with horseradish peroxidase-conjugated streptavidin (1:200; Beijing Zhongshan Biotech Co., Ltd) at 37°C for 30 min. After washing in PBS three times (3 min each), reactions were visualized by treating sections with 0.04% DAB and 0.03% H_2_O_2_ at room temperature for 8–12 min. After washing in water, sections were counterstained using hematoxylin and mounted with resin. Control reaction mixtures contained PBS instead of the TUNEL reagents. Positive-control sections were pretreated with DNase I for 10 min followed by TUNEL staining. Cells with blue granules in the nucleus were regarded as TUNEL-positive.

### Dual immunohistochemical staining

Dual immunohistochemical staining to detect TUNEL-positive and TLR3-positive cells was performed as follows: First, TUNEL staining was performed using the blue-black BCIP/NBT color reagent (Zymed Histostain-Dskit) at 37°C for 20 min. After the primary staining sequence, slides were rinsed in TBS and incubated in a double-stain blocking solution for 3 min (Dako cytomation) and then rinsed in TBS. The anti-TLR3 antibody (diluted 1:100) was applied to the tissue sections at 37°C for 60 min and incubated at 4°C overnight. A secondary antibody against goat anti-rabbit IgG conjugated to horseradish peroxidase (PV-6002) was added for 30 min at 37°C followed by rinsing in TBS. Sections were developed using liquid DAB-plus for 10 min, rinsed in TBS, stained with Mayer’s hematoxylin for 10 s, and mounted in aqueous mounting medium. The nuclei of apoptotic cells were stained blue-black (BCIP/NBT), and TLR3 in the cytoplasm was stained brown (DAB).

### Statistical analysis

Statistical analysis was performed using SPSS 19.0 for Windows. The statistical significance of the differences in data between cancer tissues and adjacent nontumor tissues was assessed using Wilcoxon signed-rank tests. The Spearman rank correlation test was performed to determine the association of HCC-related markers. TUNEL, MVD, and EPCs were analyzed using histograms and linear regression analysis, and *P* < 0.05 was considered significant. Univariate survival analyses were performed to evaluate the prognostic significance of TLR3 expression. Curves for overall survival (OS) were generated according to the Kaplan–Meier method, and differences were analyzed by applying the log-rank test to univariate survival analysis.

## Results

### TLR3 expression and localization in HCC and adjacent nontumor tissues

**Figure 1:**
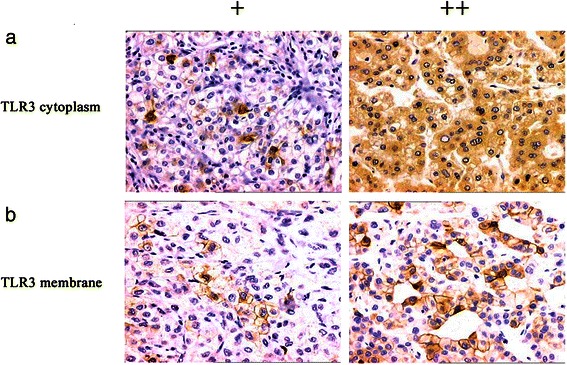
Immunohistochemical analysis of TLR3 expression and localization in HCC tissues. TLR3 was detected in the cytoplasm (**a**) and membrane (**b**). Magnification × 200.

Immunohistochemical analyses of TLR3 expression were conducted using tissues collected from 85 patients with HCC along with the respective adjacent uninvolved tissue as well as 30 non-HCC tissue specimens (Figure 1 and Table 2). TLR3 expression in the cytoplasm or membrane was detected in 58.8% (50/85), 67.1% (57/85), and 80.0% (24/30) of the tumor, normal, and non-HCC sections, respectively. There was no significant difference in TLR3 expression between HCC and adjacent tissues (*χ*^2^ = 1.236, *P* > 0.05) or between adjacent and nontumor tissues (*χ*^2^ = 1.783, *P* > 0.05). The frequency of TLR3 expression in HCC samples was significantly lower compared with that of nontumor tissues (*χ*^2^ = 4.334, *P* < 0.05).

**Table 2:** **TLR3 expression in HCC, adjacent tissues, and other liver tissues**

Sample	N	TLR3 expression			Positive rate (%)
		−	+	++	
HCC	85	35	22	28	58.8^ab^
Adjacent	85	28	23	34	67.1^c^
Nontumor	30	6	11	13	80.0

(^a^*P* > 0.05, HCC vs Adjacent; ^b^*P* < 0.05, HCC vs Nontumor; ^c^*P* > 0.05, Adjacent vs Nontumor).

### Correlation of TLR3 expression and localization with HCC histological grade

The frequencies of TLR3 expression in HCC tissues according to histological grade were as follows: G1 77.78% (7/9), G2 62.86% (22/35), and G3 51.22% (21/41). Thus, TLR3 expression was more frequent in well-differentiated HCC tissues. There were no significant associations between expresson of TLR3 in the membrane and in both the membrane and cytoplasm and HCC histological grade (*χ*^2^ = 1.057, *P* = 0.590; *χ*^2^ = 2.017, *P* = 0.365; respectively). Conversely, there was a significant positive relationship between the expression of TLR3 in the cytoplasm and HCC histological grade (*χ*^2^ = 8.354, *P* = 0.015) (Table 3).

**Table 3:** **Association of TLR3 expression and intracellular localization with the stages of HCC**

Item	Positive (N)	TLR3 expression pattern		
		M	P	M/P
G1	7	0	7	0
G2	22	4	11	7
G3	21	4	10	7

### The association of TLR3 expression with HBV infection of patients with HCC

Patients’ serum levels of HBsAg and HBcAg were determined using an ELISA. There was a significant positive correlation between the expression of TLR3 in the cytoplasm of HCC cells and serum levels of HBsAg (*χ*^2^ = 24.299, *P* < 0.001; r = 0.551, *P* < 0.001) (Table 4). Conversely, there was a significant negative correlation between the expression of TLR3 in the membrane or both the membrane and cytoplasm of cells in HCC tissues with the levels of serum HBsAg (*P* > 0.05). However, there was no significant correlation between TLR3 expression and levels of serum HBcAg (*P* > 0.05).

**Table 4:** **The association of TLR3 expression with HBV infection of patients with HCC**

Item	N	TLR3 expression pattern			Statistical results
		M^a^	P^b^	MP^c^	
HBsAg	+23	2	17	0	a: *χ*^2^ = 0.028, *P = 0.617* > 0.05; *r* = −0.019, *P = 0.869* > 0.05
					b: *χ*^2^ = 24.299, *P = 0.000* < 0.001; *r* =0.551, *P = 0.000* < 0.001
	−62	6	11	14	c: *χ*^2^ = 3.527, *P = 0.053* > 0.05; *r* = −0.210, *P = 0.062* > 0.05
HBcAg	+13	1	5	2	a: *χ*^2^ = 0.044, *P = 0.656* > 0.05; *r* = −0.023, *P = 0.837* > 0.05
					b: *χ*^2^ = 0.257, *P = 0.445* > 0.05; *r* = −0.057, *P = 0.618* > 0.05
	−72	7	23	12	c: *χ*^2^ = 0.550, *P = 0.350* > 0.05; *r* =0.083, *P = 0.465* > 0.05

^a^TLR3 M expression compared with HBsAg or HBcAg serum levels. ^b^TLR3 P expression compared with HBsAg serum levels. ^c^TLR3 M/P expression compared with HBsAg serum levels. Abbreviations: M, membrane; P, cytoplasm; MP membrane and cytoplasm.

### Immunohistochemical analysis of TLR3, TRIF, NF-κB, and IRF3 expression in HCC tissues

**Figure 2:**
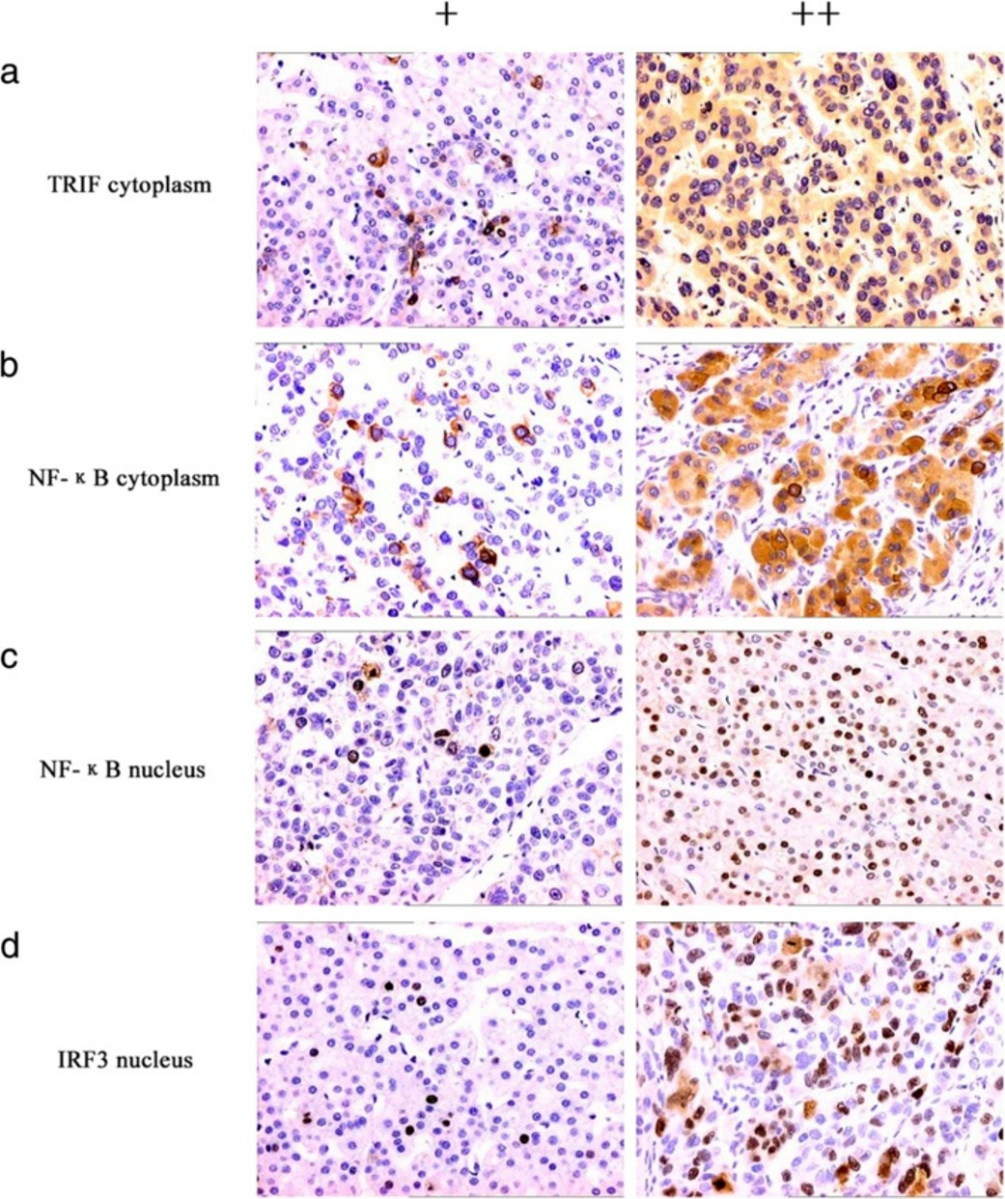
Immunohistochemical analysis of TRIF, NF-κB, and IRF3 expression and localization in HCC tissues. TRIF was detected in the cytoplasm (**a**), NF-κB was detected in the cytoplasm or nucleus (**b**, **c**), and IRF3 was detected in the nucleus (**d**). Magnification × 200.

TRIF was detected in the cytoplasm of cells in 69.4% (59/85) of HCC tumor sections. NF-κB was detected in the cytoplasm or nucleus in 63.5% (54/85) of HCC tumor sections. IRF3 was expressed in the nucleus in 52.9% (45/85) of HCC tumor sections (Figure 2). The frequency of TLR3 expression in HCC tumor sections correlated positively with those of TRIF (γ = 0.322, *P <* 0.01), NF-κB (γ = 0.264, *P <* 0.05), and IRF3 (γ = 0.317, *P <* 0.01) (Table 5).

**Table 5:** **Correlation between expression of TLR3, TRIF, NF-κB, and IRF3 in HCC tissues**

Item	N	TLR3 expression			*χ*^2^test		Spearman correlation analysis	
		−	+	++	*χ* ^2^	*P*	γ	*P*
TRIF					16.975	0.002	0.322	0.003
−	26	15	3	8				
+	25	14	8	3				
++	34	6	11	17				
NF-κB					16.444	0.002	0.264	0.014
−	31	18	6	7				
+	31	9	14	8				
++	23	8	2	13				
IRF3					9.568	0.048	0.317	0.003
−	40	22	11	7				
+	24	8	5	11				
++	21	5	6	10				

### The association of TLR3 signaling-pathway protein expression and apoptosis in HCC tissues

**Figure 3:**
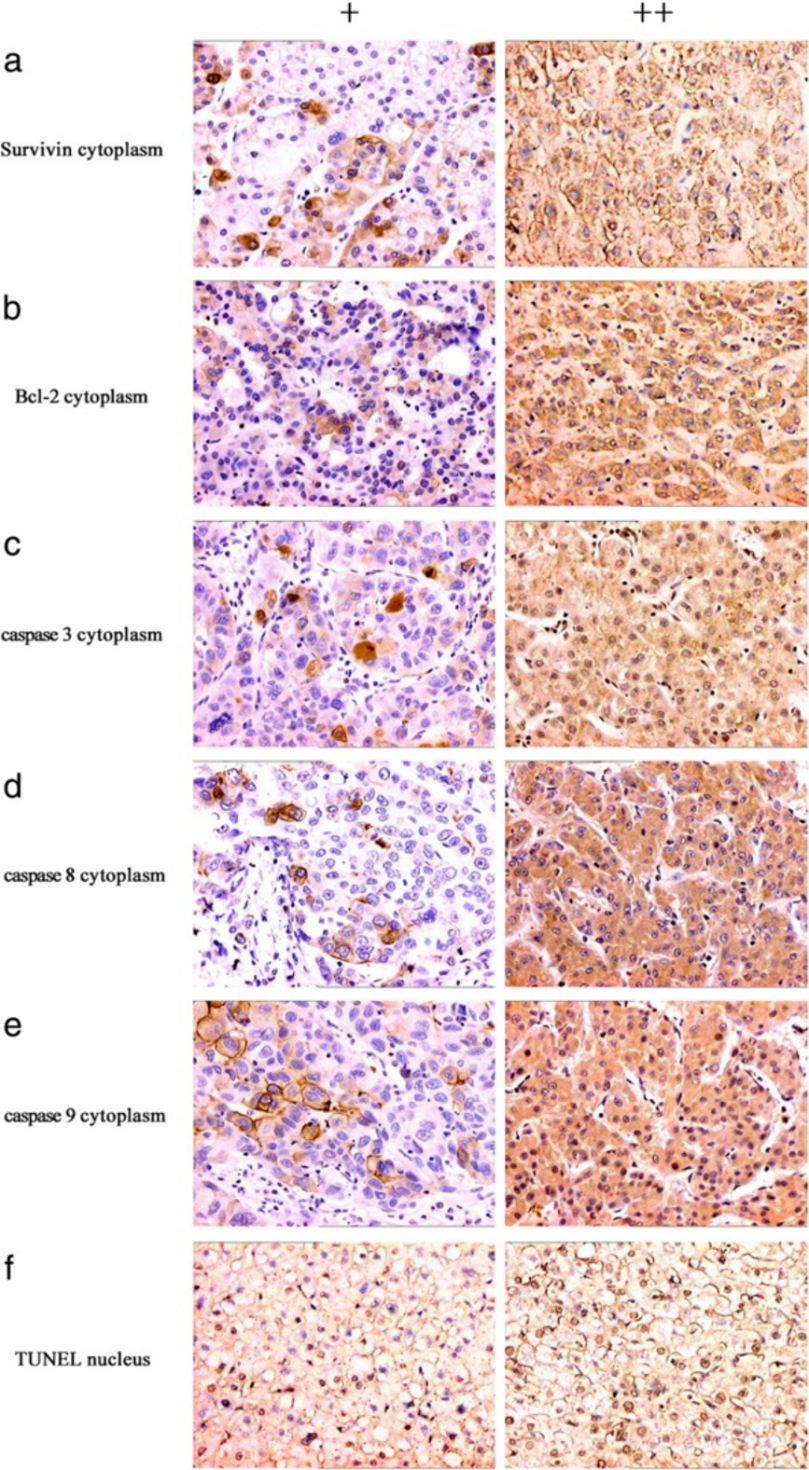
Immunohistochemical analysis of survivin, Bcl-2, and caspase expression and TUNEL analysis of apoptosis in HCC tissues. Survivin (**a**), Bcl-2 (**b**), and caspase 3, 8 and 9 were detected in the cytoplasm (**c**, **d** and **e**). Apoptotic nuclei detected using the TUNEL assay are brownish-yellow (**f**). Magnification × 200.

Using immunohistochemistry, we detected the cytoplasmic expression of the apoptosis-related proteins survivin, Bcl-2, and caspases 3, 8, and 9 (Figure 3), which were expressed in 48.2% (41/85), 38.8% (33/85), 71.8% (61/85), 68.2% (58/85) and 57.6% (49/85), respectively, of HCC tissue samples. Survivin expression correlated negatively with those of TLR3 (γ = −0.360, *P <* 0.01), TRIF (γ = −0.234, *P <* 0.05), IRF3 (γ = −0.286, *P <* 0.01), and NF-κB (γ = −0.246, *P <* 0.05). Bcl-2 expression correlated negatively with that of TLR3 (γ = −0.369, *P <* 0.01), TRIF (γ = −0.288, *P <* 0.01), and IRF3 (γ = −0.236, *P <* 0.05) in HCC tissue samples (Table 4). The frequencies of caspases 3, 8, and 9 expression correlated positively with those of TLR3 signaling proteins (*P <* 0.01 or *P <* 0.05) (Table 6).

**Table 6:** **Correlation between the expression of TLR3 signaling-pathway proteins and apoptosis-related proteins with apoptosis in HCC tissues**

Item	N	TLR3			Spearm		TRIF			Spearman		IRF3			Spearman		NF-κB			Spearman	
		expression			analysis		expression			analysis		expression			analysis		expression			analysis	
		−	+	++	γ	*P*	−	+	++	γ	*P*	−	+	++	γ	*P*	−	+	++	γ	*P*
Survinvin					−0.360	0.001				−0.234	0.031				−0.286	0.008				−0.246	0.023
−	44	12	10	22			9	13	22			13	18	13			12	17	15		
+	23	12	8	3			10	6	7			15	4	4			9	8	6		
++	18	11	4	3			7	6	5			12	2	4			10	6	2		
Bcl-2					−0.369	0.001				−0.288	0.008				−0.236	0.030				−0.085	0.438
−	52	16	13	23			14	10	28			21	14	17			17	19	16		
+	16	6	5	5			6	5	5			8	5	3			8	6	2		
++	17	13	4	0			6	10	1			11	5	1			6	6	5		
caspase 3					0.420	0.000				0.225	0.038				0.300	0.005				0.348	0.001
−	24	15	5	4			11	10	3			15	6	3			13	8	3		
+	20	10	9	1			3	6	11			13	2	5			9	8	3		
++	41	10	8	23			12	9	20			12	16	13			9	15	17		
caspase 8					0.310	0.004				0.448	0.000				0.367	0.001				0.279	0.010
−	27	16	4	7			18	3	6			19	4	4			15	7	5		
+	23	11	8	4			1	17	5			12	7	4			8	10	5		
++	35	8	10	17			7	5	23			9	13	13			8	14	13		
caspase 9					0.249	0.022				0.312	0.004				0.275	0.011				0.616	0.000
−	36	19	10	7			15	13	8			24	7	5			27	9	3		
+	25	8	8	9			8	4	13			8	7	10			3	21	1		
++	24	8	4	12			3	8	13			8	10	6			4	1	19		
AI					0.483	0.000				0.566	0.000				0.548	0.000				0.438	0.000
≤10%	33	21	4	8			18	10	5			26	4	3			19	9	5		
11% - 20%	37	13	15	9			8	12	17			14	14	9			11	19	7		
>20%	15	1	3	11			0	3	12			0	6	9			1	3	11		

**Figure 4:**
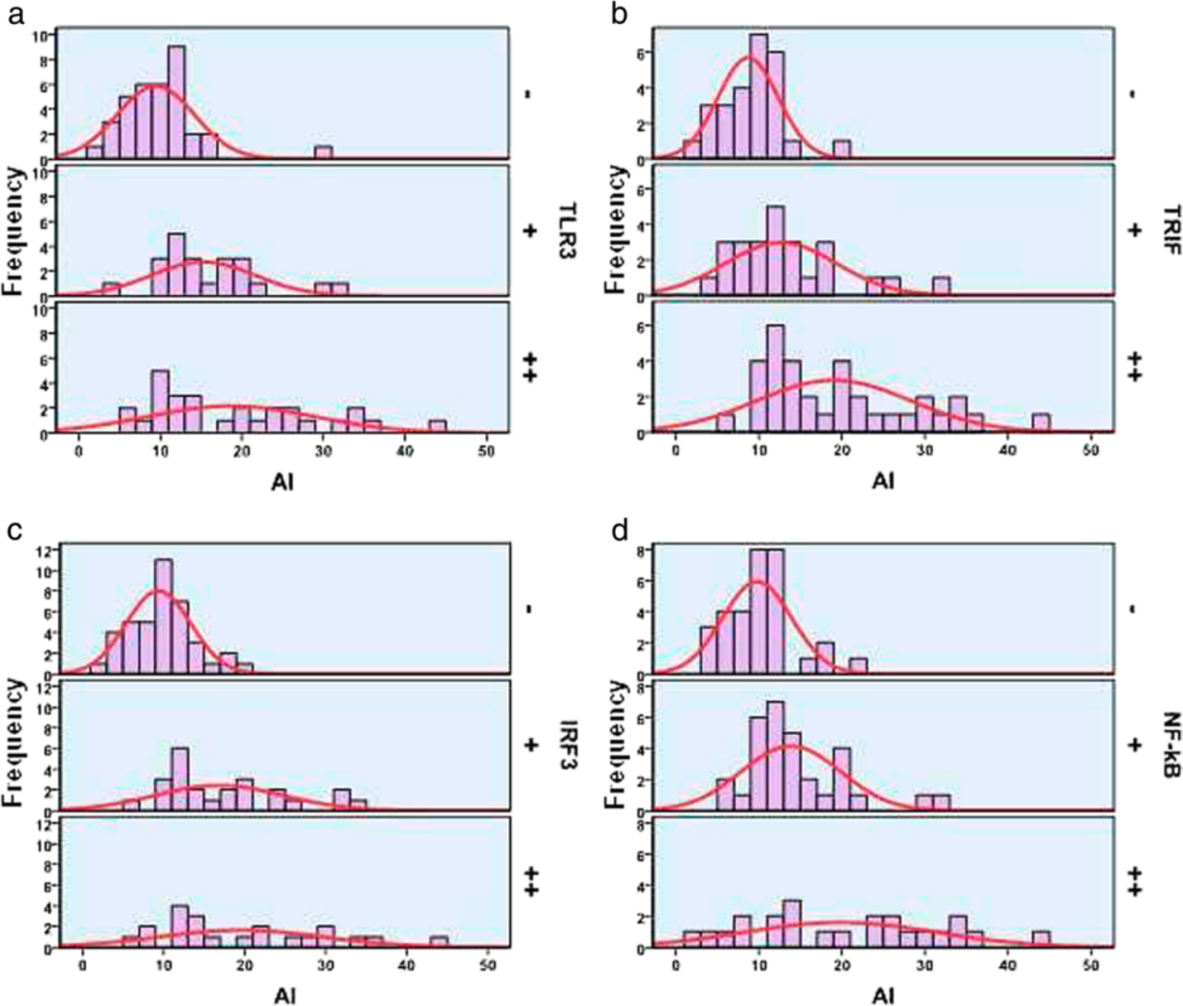
The relationship between AI and the expression of TLR3 (**a**), TRIF (**b**), IRF3 (**c**), and NF-κB (**d**).

The results of TUNEL assays (AI) in HCC tissues were 2–43% (mean = 13.98%). The 85 HCC tissues were divided according to AI values into the groups as follows: ≤10%, 11%–20%, and >20%. Spearman correlation analysis shows that AI correlated positively with the frequencies of expression of TLR3 (γ = 0.483, *P <* 0.01), TRIF (γ = 0.566, *P <* 0.01), IRF3 (γ = 0.548, *P <* 0.01), and NF-κB (γ = 0.438, *P <* 0.01) (Figure 4, Table 6).

**Figure 5:**
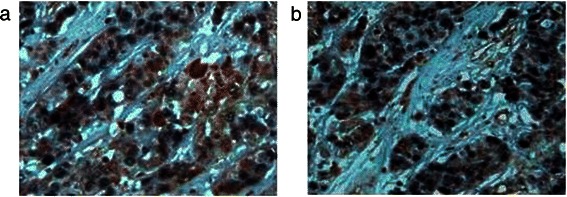
Dual immunohistochemical analysis of apoptosis and TLR3 expression in HCC tissue. (**a**) In well-differentiated HCC tissue, the nuclei were TUNEL-positive and TLR3 was overexpressed at equal levels in the cytoplasm and membrane. (**b**) The nuclei of poorly differentiated HCC cells were TUNEL-positive, and these cells expressed relatively lower levels of cytoplasmic TLR3. Magnification × 400.

For more precise localization, dual immunohistochemical analyses were performed to detect TUNEL activity and TLR3 expression. TLR3-positive and TUNEL-positive signals were localized to the tumor cell cytoplasm or membrane and to the nucleus, respectively (Figure 5). The two markers correlated positively in situ (Figure 5).

### Correlation of TLR3 expression with apoptosis (TUNEL assay) and serum levels of HBV antigens

When TLR3 was expressed in the cytoplasm, the TUNEL data correlated positively with HBsAg levels in serum (*χ*^2^ = 9.420, *P* = 0.003; r = 0.614, *P* = 0.001) and negatively with HBcAg levels in serum (*P* > 0.05). When TLR3 was expressed in the membrane or in the membrane and cytoplasm, TUNEL positivity correlated negatively with HBsAg and HBcAg levels (*P* > 0.05).

### The association of the expression of TLR3-pathway-signaling proteins with those of proliferation-related proteins Ki67 and cyclinD1 in HCC tissues

**Figure 6:**
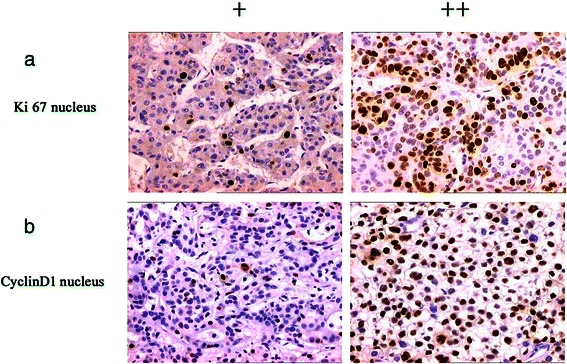
Analysis of Ki67 and Cyclin D1 expression and localization in HCC cells. (**a**) Ki67 was detected in the nucleus. CyclinD1 exhibited nucleus staining (**b**). Magnification × 200.

The expressions of Ki67 and cyclin D1 were detected using immunohistochemistry (Figure 6). Expression was localized to the nucleus with the frequencies as follows: Ki67 61.2% (52/85) and cyclin D1 57.6% (49/85). The frequency of Ki67 expression correlated negatively with those of TLR3 (γ = −0.276, *P <*0.05), TRIF (γ = −0.215, *P <*0.05), IRF3 (γ = −0.281, *P <*0.01), and NF-κB (γ = −0.265, *P <*0.05). Cyclin D1 expression correlated negatively with those of TLR3 (γ = −0.269, *P <*0.05), TRIF (γ = −0.219, *P <*0.05), IRF3 (γ = −0.292, *P <* 0.01), and NF-κB (γ = −0.262, *P <*0.05) (Table 7).

**Table 7:** **Correlation between the expression of TLR3 signaling-pathway proteins with those of Ki67, cyclin D1, MMP-2, CD34 and analysis of EPCs in HCC tissues**

Item	N	TLR3			Spearman		TRIF			Spearman		IRF3			Spearman		NF-κB			Spearman	
		expression			analysis		expression			analysis		expression			analysis		expression			analysis	
		−	+	++	γ	*P*	−	+	++	γ	*P*	−	+	++	γ	*P*	−	+	++	γ	*P*
Ki-67					−0.276	0.011				−0.215	0.048				−0.281	0.009				−0.265	0.014
−	33	12	4	17			8	9	16			10	12	11			9	12	12		
+	36	12	15	19			12	7	17			19	9	8			12	14	10		
++	16	11	3	2			6	9	1			11	3	2			10	5	1		
CyclinD1					−0.269	0.013				−0.219	0.044				−0.292	0.007				−0.262	0.016
−	36	11	7	18			10	6	20			11	13	12			12	11	13		
+	28	12	10	6			9	11	8			17	5	6			11	12	5		
++	21	12	5	4			7	8	6			12	6	3			8	8	5		
MMP-2					−0.378	0.000				−0.294	0.006				−0.196	0.073				−0.248	0.022
−	37	10	9	18			8	8	21			15	9	13			11	12	14		
+	25	9	8	8			9	7	9			10	12	3			8	10	7		
++	23	16	5	2			9	10	4			15	3	5			12	9	2		
MVD					−0.583	0.000				−0.560	0.000				−0.527	0.000				−0.484	0.000
≤120	23	3	4	16			1	4	18			16	7	2			2	8	13		
121–240	37	11	17	9			9	14	14			15	9	13			12	19	6		
>240	25	21	1	3			16	7	2			22	2	1			17	4	4		
EPCs count					−0.544	0.000				−0.458	0.000				−0.277	0.010				−0.345	0.001
≤5	54	13	18	23			10	14	30			21	17	16			14	23	17		
6 - 10	22	14	4	4			8	11	3			10	7	5			10	7	5		
>10	9	8	0	1			8	0	1			9	0	0			7	1	1		

### The association of the expression of TLR3 signaling-pathway proteins with those of CD34 and MMP-2 in HCC tissues

**Figure 7:**
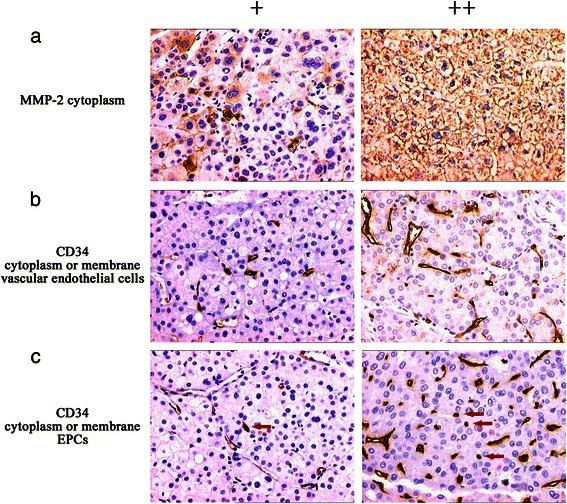
MMP-2 and CD34 expression and location in HCC cells. (**a**) MMP-2 was detected in the cytoplasm. (**b**) CD34 was detected in the membrane or cytoplasm of vascular endothelial cells. (**c**) CD34 was detected in the membrane or cytoplasm of in EPCs (red arrows). Magnification × 200.

**Figure 8:**
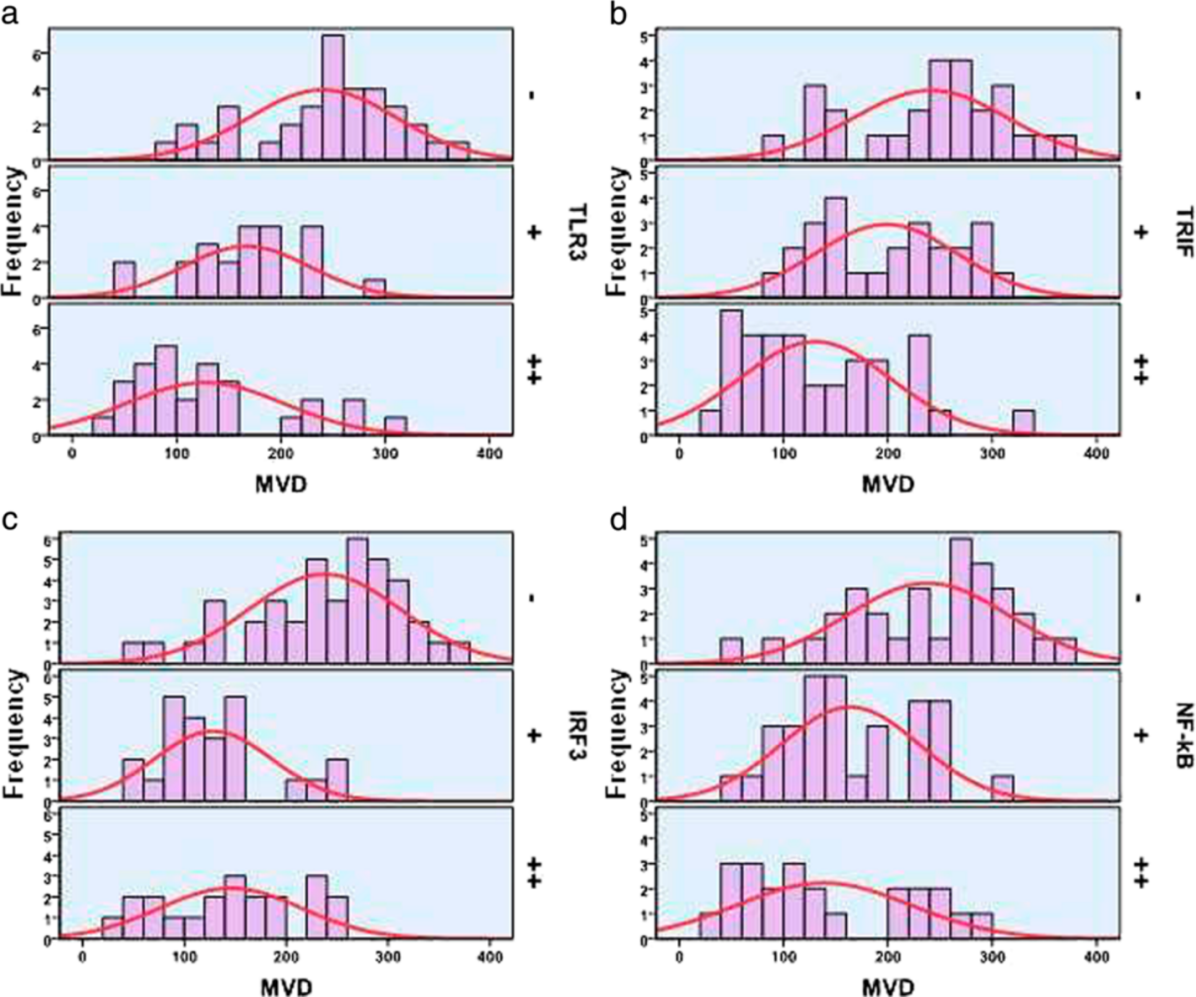
The relationship between MVD and the expression of TLR3 (**a**), TRIF (**b**), IRF3 (**c**), and NF-κB (**d**).

**Figure 9:**
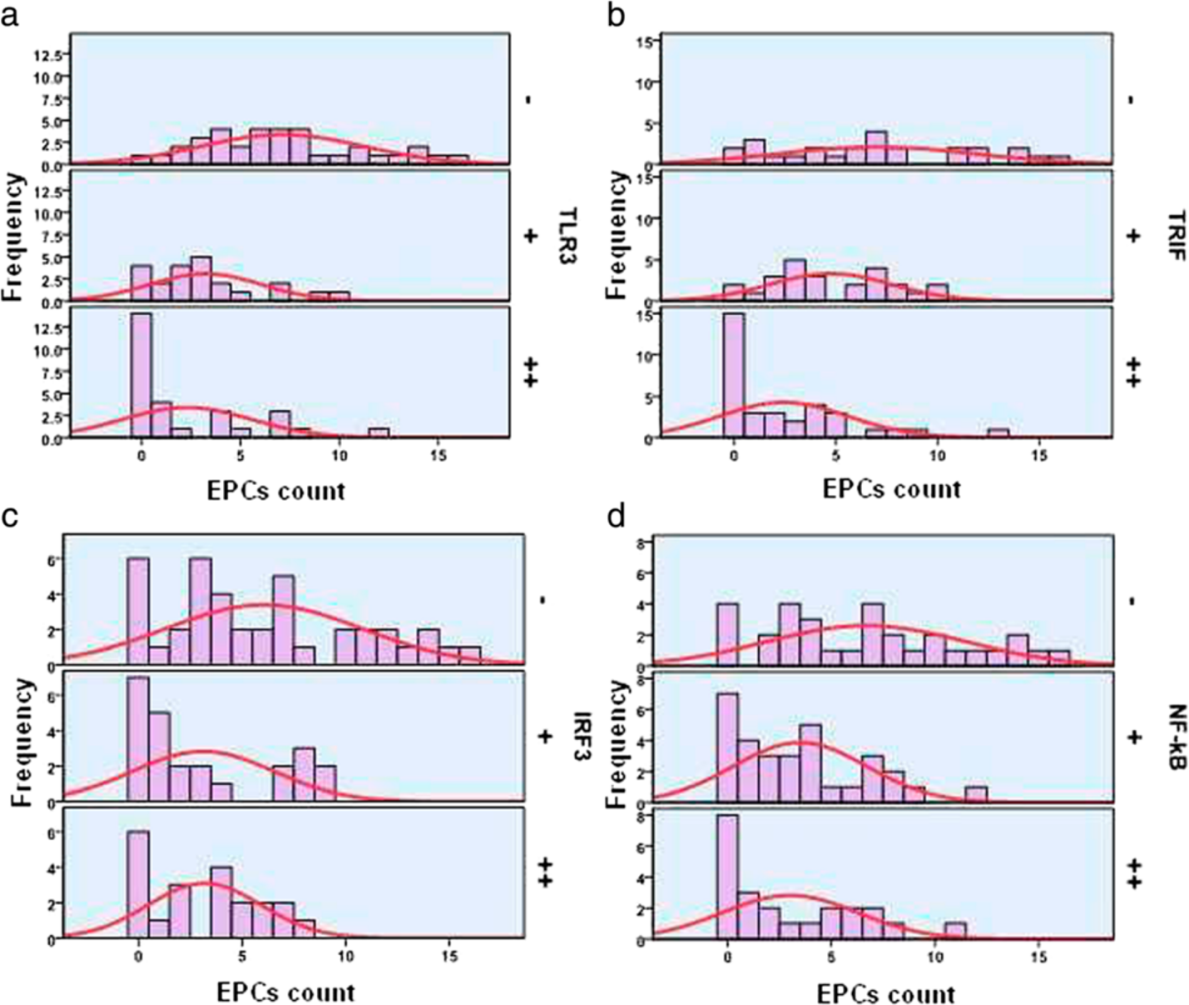
The relationship between the number of EPCs and the expression of TLR3 (**a**), TRIF (**b**), IRF3 (**c**), and NF-κB (**d**).

Immunohistochemical analysis detected MMP-2 expression in the cytoplasm (Figure 6) of cells in 56.5% (48/85) of samples. MMP-2 expression levels correlated negatively with those of TLR3 (γ = −0.378, *P <*0.01), TRIF (γ = −0.294, *P <*0.01), and NF-κB (γ = −0.248, *P <* 0.05) (Table 7). CD34 was detected in the membranes or cytoplasm of vascular endothelial cells and EPCs (Figure 7). The average MVD of the 85 HCC tissue samples was 183.89 mm^2^. The samples were divided into grades as follows: ≤120, 121–240, and >240. Spearman correlation analysis shows that MVD correlated negatively with the frequency of expression of TLR3 (γ = −0.583, *P <* 0.01), TRIF (γ = −0.560, *P <* 0.01), IRF3 (γ = −0.527, *P <* 0.01), and NF-κB (γ = −0.484, *P <* 0.01) (Figure 8, Table 8). Moreover, the average EPC count of the 85 HCC samples was 0–16 (mean = 4.52). The samples were divided into grades as follows: ≤5, 6–10, and >10. Spearman correlation analysis shows that the EPC count correlated negatively with the frequencies of expression of TLR3 (γ = −0.544, *P <* 0.01), TRIF (γ = −0.458, *P <* 0.01), IRF3 (γ = −0.277, *P <* 0.05), and NF-κB (γ = −0.345, *P <* 0.01) (Figure 9, Table 8).

**Table 8:** **Correlation of the expression pattern of TLR3 with apoptosis in HCC tissues and serum levels of HBV antigens**

Item	N	P^a^		M^b^		M/P^c^		Statistical results
		TUNEL		TUNEL		TUNEL		
		−/+	++	−/+	++	−/+	++	
HBsAg								a: *χ*^2^ = 9.420, *P = 0.003* < 0.05; *r* =0.614, *P = 0.001 <* 0.05
+	23	4	13	2	0	0	0	b: *χ*^2^ = 0.381, *P = 0.750* > 0.05; *r* = −0.218, *P = 0.604* > 0.05
−	62	9	2	5	1	12	2	c: *χ*^2^ = 3.949, *P = 0.214* > 0.05; *r* =0.531, *P = 0.051 >* 0.05
HBcAg								a: *χ*^2^ = 3.147, *P = 0.124 >* 0.05; *r* =0.355, *P = 0.082 >* 0.05
+	13	1	4	1	0	2	0	b: *χ*^2^ = 0.163, *P = 0.875* > 0.05; *r* = −0.143, *P = 0.736* > 0.05
−	72	13	10	6	1	10	2	c: *χ*^2^ = 0.321, *P = 0.547 >* 0.05; *r* =0.152, *P = 0.605 >* 0.05

^a^TLR3 P expression, positive TUNEL reactions compared with HBsAg or HBcAg levels. ^b^TLR3 M expression, positive TUNEL reactions compared with HBsAg or HBcAg levels. ^c^TLR3 M/P expression, positive TUNEL reactions compared with HBsAg or HBcAg levels.

### The association of the expression of TLR3 signaling-pathway proteins with survival of patients with HCC

**Figure 10:**
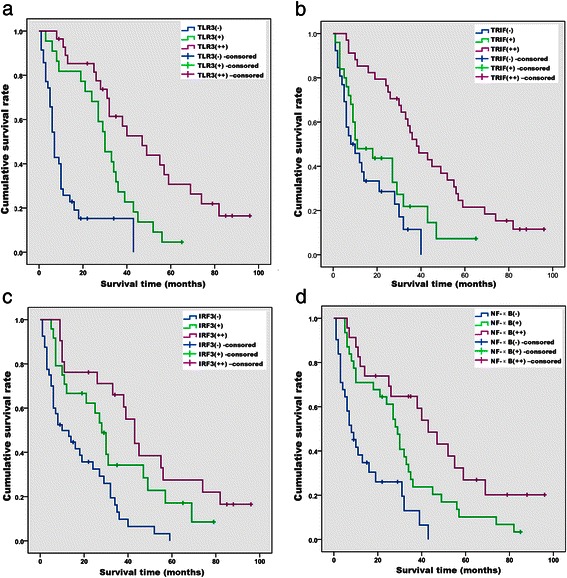
Kaplan–Meier survival curves stratified according to the expression of TLR3 (**a**), TRIF (**b**), IRF3 (**c**), and NF-κB (**d**) (*n* = 76).

The follow-up and loss rates of patients were 89.4% (76/85) and 10.6% (9/85), respectively. The follow-up period ranged from 1–96 months (median = 26 months). Among the 76 patients followed after their discharge, 71 died of HCC and one died of other causes. The 1-, 3-, and 5-year media overall survival (OS) rates for these 76 patients were 56.6% (43/76), 27.6% (21/76), and 10.5% (8/76), respectively. The survival rates of patients increased significantly as a function of increased expression levels of TLR3, TRIF, IRF3, and NF-κB as follows (expression levels followed by survival rates): 1. TLR3 (−), (+), and (++): 0% (0/35), 4.5% (1/22), and 25.0 (7/28), respectively (log-rank = 43.187, *P* < 0.01) (Figure 10a). 2. TRIF (−), (+), and (++): 0% (0/26), 4.0% (1/25), and 20.6% (7/34), respectively (log-rank = 21.867, *P* < 0.01) (Figure 10b). 3. IRF3 (−), (+), and (++): 0% (0/40), 12.5% (3/24), and 23.8 (5/21), respectively (log-rank = 19.818, *P* < 0.01) (Figure 10c). 4. NF-κB (−), (+), and (++): 0% (0/31), 9.7% (3/31), and 21.7 (5/23), respectively (log-rank = 23.375, *P* < 0.01) (Figure 10d).

## Discussion

HCC is the sixth most common malignancy of humans, accounting for approximately 90% of primary liver cancers [18]. Hepatocarcinogenesis is complex and characterized by myriad molecular abnormalities [19]. Over the past 10 decades, the molecular mechanism of HCC has been extensively investigated. However, our knowledge of the pathogenesis of this disease is insufficient for the purposes of prevention, early diagnosis, and treatment. Increasing evidence indicates that TLR3 is an important modulator of HCC progression and a potential target for novel immunotherapy [20].

TLR3 is an endosomal receptor for double-stranded RNA and is expressed by several subsets of immune cells, including dendritic cells [21] and natural killer (NK) cells [22]. TLR3 is expressed by fibroblasts [23], lung epithelial cells [24], hepatocytes [25], and several types of tumor cells. For example, Yoneda [26] found that *TLR3* mRNA was expressed in HCC tissues as well as in nontumor tissues. Using immunocytochemistry, TLR3 was detected in 52.7% of HCC tissues. Although cell-surface stimulation of TLR3 with poly (I:C) does not affect cell viability, it does increase NF-κB levels. In contrast, cytoplasmic stimulation with transfected poly (I:C) induced apoptosis, which was accompanied by down-regulation of antiapoptotic proteins. In the present study, we used immunohistochemistry and found that TLR3 was expressed in 58.8% (50/85) of HCC tissues, which is slightly higher than previously reported, and that TLR3 was localized to the membrane and cytoplasmic. TLR3 was expressed more frequently in tumor tissues compared with adjacent tissues (67.1%) and tissues from subjects without HCC (80.0%). Further, TLR3 expression was stronger in well-differentiated HCC tissues compared with poorly differentiated HCC tissues, and there was a significant positive relationship between the cytoplasmic expression of TLR3 and HCC histological grade, which suggests that down-regulation of TLR3 may disrupt the regulation of cell proliferation and homeostasis to promote malignant transformation.

TLR3 signaling depends solely on the TLR TIR domain, which recruits the adaptor-inducing IFN-β (TRIF) adapter protein. This leads to activation of the transcription factors NF-κB and IRF3 and induces the antiviral interferon response [6]. Further, TRIF exhibits proapoptotic activity, suggesting that TLR3 signaling triggers the cell death pathway [27]. In the present study, we detected the TLR3 signaling-pathway proteins TRIF, NF-κB, and IRF3 in 69.4% (59/85), 63.5% (54/85), and 52.9% (45/85) of the HCC samples, respectively. Moreover, their expression correlated positively with that of TLR3. Therefore, the increased frequency of TLR3 expression may affect the proliferation and apoptosis of HCC cells through multiple signaling pathways.

TLR3 is unique among TLRs, because it signals through TRIF (TIR domain-containing adaptor-inducing interferon-β), not through MyD88, and may activate the inflammatory or apoptotic pathways. The inflammatory pathway leads to NF-κB activation, whereas the apoptotic pathway, believed to be mediated by Rip3, leads to caspase-8 activation [28]. Our results suggest that TLR3-TRIF-IRF3 or the TRIF-NF-κB signaling pathway is activated in HCC cells in the majority of tissue samples analyzed here.

Apoptosis is a complex process that is mainly mediated through the Fas ligand/Fas pathway as well as a mitochondrial pathway [29]. To examine the mechanisms of TLR3-induced apoptosis in HCC cells, we studied the association of TLR3 expression by HCC cells with the expression of apoptosis-related proteins Bcl-2, survivin, and caspases 3, 8, and 9. Bcl-2 localizes to the inner mitochondrial membrane [30] and is important for cell survival and its antiapoptotic effects. Survivin is critically required for inhibiting apoptosis and ensuring normal cell division in the G2/M phase of the cell cycle and is abundantly expressed in every human tumor compared with normal tissues. Survivin inhibits apoptosis by inhibiting activated caspases [31]. The caspases, particularly caspase-3, act downstream of the Bax/Bcl-2 control and play a key role in the execution of apoptosis [32]. In the present study, the expression level of TLR3 correlated negatively with those of Bcl-2 and survivin, and correlated positively with those of caspases 3, 8, and 9, indicating that activation of TLR3 is related to the stimulation of apoptosis.

Detection here of TLR3 in the cytoplasm and membranes of HCC cells was accompanied by activation of the components of the Fas ligand/Fas and mitochondrial apoptotic pathways, suggesting that TLR3 promotes apoptosis of HCC cells through these pathways. Moreover, we demonstrate a significant positive relationship between the expression of the TLR3 signaling-pathway components TRIF, IRF3, and NF-κB as well as those of caspases 3, 8, and 9. TUNEL staining shows that the AI correlated positively with the expression of TRIF, IRF3, and NF-κB. Our data identify an association of TLR3 with overexpression of caspases 3, 8, and 9 and suggest that activation of TLR3 plays an important role in apoptosis in HCC through the Fas ligand/Fas and mitochondrial pathways.

We demonstrate here a significant positive relationship between the cytoplasmic expression of TLR3 and the presence of HBsAg in serum. Moreover, when TLR3 was expressed in the cytoplasm, TUNEL positivity correlated with the detection of HBsAg in serum, indicating that the synthesis of viral dsRNA was upregulated and activated in TLR3, which in turn increased the population of interstitial immunoreactive cells and induced the production of inflammatory cytokines.

The cytokine interleukin (IL)-1β is a key mediator of the inflammatory response and is implicated in the pathophysiology of acute and chronic inflammation. Poly (I:C) stimulation of macrophages induces pro-IL-1β processing via a Toll/IL-1R domain-containing adaptor-inducing interferon-β-dependent signaling pathway initiated by TLR3 [33]. Boulton et al. found that IL-1β administered parenterally to rats 0 and 12 h after partial hepatectomy significantly reduced the incorporation of bromodeoxyuridine into hepatocytes at 18 h, indicating that nonparenchymal cells isolated from regenerating rat liver express IL-1. These findings support the hypothesis that IL-1 plays a role in suppressing hepatocyte proliferation and terminating the surge of DNA synthesis induced after partial hepatectomy [34]. In contrast, decreased induction of IL-1β mRNA synthesis in TLR3-deficient mice leads to the proliferation of hepatocytes [33]. These findings suggest that TLR3 activation inhibits cell proliferation, which supports the conclusion that IL-1β mediates antitumor activity. Consistent with these findings, we show here a negative correlation between the expression of TLR3 and Ki67 as well as with cyclin D1. Moreover, we demonstrate a significant negative relationship between the expression of TLR3 pathway-signaling proteins and that of Ki67 or cyclin D1.

Angiogenesis plays an important role in the malignant transformation, growth, and metastasis of parenchymal tumors. Tumor angiogenesis is regulated by angiogenic factors generated and secreted by tumor cells. HCC is a highly vascularized tumor that requires the formation of numerous blood vessels to receive a sufficient blood supply required for growth and proliferation. Thus, angiogenesis is a crucial process in the development of HCC. MMP-2 is associated with tumor malignancy [35]. EPCs are considered the primary resource for postnatal vasculogenesis and are detected in peripheral blood, cord blood, spleen, vessel walls, and heart and skeletal muscles [36]. CD34^+^ EPCs express a range of diverse surface markers and contain progenitor cells that are capable of differentiating into endothelial and osteogenic lineages under the appropriate conditions [37].

Preclinical studies show that despite their heterogeneity, human CD34^+^ EPCs stimulate neovascularization in ischemic myocardium by increasing capillary density and improving function in models of acute and chronic myocardial ischemia [38]. Yang et al. [39] found that treatment of EPCs with the TLR3 agonist poly (I:C) upregulates the expression of the cytokines IL-1β, IL-6, IL-8, TNF-α, IFN-α, and IFN-β, indicating that EPCs express functional TLR3. Poly (I:C) impairs cell proliferation by inducing cell cycle progress inhibition and cell apoptosis via TLR3 in EPCs. For example, Guo et al. [40] found that rat aortic ring outgrowth and endothelial cell tube formation are suppressed after treatment with dsRNA and that dsRNA triggers apoptosis of the MHCC97H, SMMC-7721, and HUVEC cell lines and inhibits cell migration. Our findings are consistent with studies showing that MVD and EPCs correlate negatively with the expression of TLR3 signaling-pathway proteins in HCC. Moreover, MMP-2 expression correlated inversely with the expression of TLR3 signaling-pathway proteins in HCC. These findings suggest that TLR3 activation not only affects the tumor microenvironment by suppressing angiogenesis but also directly inhibits tumor cell invasion.

The activation of TLR3 was associated with malignant progression and prognosis of patients. HCC is the second most frequent cause of cancer death. Because of the development of improved treatment strategies and monitoring methods, the 5-year overall survival rate of HCC increased by 11.8% in the United States [41] and by 17.0% in Germany [42]. Our data for 1-, 3-, and 5-year survival rates were 56.6%, 27.6%, and 10.5%, respectively, and are still lower compared with those of more developed countries. Here we show that prognosis was better for patients with HCC tissues positive for TLR3 and its downstream signaling molecules TRIF, IRF3, and NF-κB, confirming that activation of TLR3 is an important factor in improving overall survival rates of patients with HCC. This may be attributed to the activation of the TLR3 pathway, which inhibited HCC cell proliferation, angiogenesis, and induced apoptosis.

## Conclusions

Our study shows that the expression of TLR3 was suppressed in more than 50% of HCC tissues of our patient population. Decreased TLR3 expression was related to tumor cell proliferation, upregulated angiogenesis, and inhibition of apoptosis, which may be associated with tumor progression and poor prognosis of patients with HCC. Therefore, the expression of TLR3 may serve as a valuable marker to estimate HCC progression, and TLR3 function may play an important role in apoptosis by inhibiting the growth and invasion of HCC cells, the production of EPCs, and angiogenesis. However, further studies are required to confirm these findings and to provide a better understanding of the mechanisms of TLR3 signaling in the development of HCC.
